# Superior accuracy of femoral bone tunnel drilling in robot-assisted anterior cruciate ligament reconstruction: a multicenter, randomized, controlled trial

**DOI:** 10.1097/JS9.0000000000002439

**Published:** 2025-05-12

**Authors:** Ling Zhang, Hansheng Hu, Wennuo Huang, Junjie Xu, Jinzhong Zhao, Wenyong Fei, Shaobai Wang

**Affiliations:** a School of Exercise and Health, Shanghai University of Sport, Shanghai, China; b Department of Orthopedics and Sports Medicine, Northern Jiangsu People’s Hospital Affiliated to Yangzhou University, Jiangsu, China; c Department of Radiological Sciences, Northern Jiangsu People’s Hospital Affiliated to Yangzhou University, Jiangsu, China; d Department of Sports Medicine, Shanghai Sixth People’s Hospital, Shanghai Jiao Tong University, Shanghai, China

**Keywords:** knee, navigation, safety, surgical robot, tunnel positioning

## Abstract

**Purpose:**

To assess the efficacy and safety for bone tunnel drilling in anatomic anterior cruciate ligament (ACL) reconstruction with the assistance of a surgical navigation robot system.

**Methods:**

A total of 79 patients were randomized to either surgical navigation robot group (robot group, *n* = 39) or traditional handheld locator group (control group, *n* = 40). The robot group underwent anatomic ACL reconstruction using a surgical navigation robot, while the control group underwent the procedure using a traditional handheld locator. Postoperative three-dimensional computed tomography was used to measure the tibial and femoral tunnel position, as well as the tibial and femoral tunnel length. The success rate of femoral tunnel positioning was defined as the proportion of cases in which the femoral tunnel was placed accurately within the ideal anatomical position.

**Results:**

The success rate of femoral tunnel positioning in the robot group was significantly higher than that in the control group (82.1% vs 50%, *P* = 0.003). The surgical time in the robot group was significantly longer than that in the control group (122.8 min ± 34.9 min vs 84.0 min ± 28.3 min, *P* = 0.05). The incidence rate of adverse events did not show statistical significance between the two groups (*P* = 0.830). There were no adverse events associated with the instruments or any serious adverse events, and no patients withdrew from the trial due to adverse events.

**Conclusions:**

The success rate for femoral tunnel positioning in anatomic ACL reconstruction was higher with surgical navigation robots compared to the traditional handheld locator. Surgical navigation robot systems are safe tools in anatomic ACL reconstruction surgery.

## Introduction

Anterior cruciate ligament (ACL) reconstruction is the primary treatment approach to improve functional stability after ACL injuries^[^1^]^. There are approximately 120 000 ACL reconstructions performed every year in the United States^[^2^]^. The failure rates in primary ACL reconstruction is an ongoing topic, with rates ranging from 3.2% to 11.1% in general cases and reported as high as 34.2% in high-risk populations such as young athletes^[^3^]^. The most common cause of ACL graft failure is a technical error involving tunnel malposition, with nonanatomic placement of the femoral tunnel accounting for up to 72% of graft failure^[^4^]^.

**HIGHLIGHTS**

- The integration of navigation systems and surgical robotics can more accurately locate and navigate bone tunnels.
- There have been no reports on the clinical use of surgical navigation robot in ACL reconstruction surgery.
- Robot-assisted ACL reconstruction offers superior accuracy in drilling the femoral tunnel compared to the traditional handheld locator procedure.
- Although an increase in surgical time associated with surgical navigation robots has been noted, it is probable that the duration of surgery will be significantly reduced as surgeons gain proficiency in robotic procedures.

Placing the tunnels in the anatomic positions is the main consideration in anatomic ACL reconstruction and is essential for successful restoration of knee function^[^5^]^. In the traditional method, surgeons use handheld locators to determine tunnel placement under an arthroscopic system^[^6,7^]^. However, factors such as visual distortion^[^8^]^, individual variability in the ACL attachment point^[^7^]^, and the learning curve of the surgeon may lead to the incorrect identification of these bony landmarks, resulting in inaccurate tunnel placement^[^9^]^. In order to improve the accuracy of femoral tunnel placement, intraoperative fluoroscopy has been introduced to determine the anatomical femoral tunnel position^[^10^]^. But notably, if the positioning during fluoroscopy is incorrect or suboptimal, it can lead to distorted or unclear images, making it challenging for the surgeon to place tunnels in the desired anatomical locations^[^10-12^]^. In addition, the use of fluoroscopically-assisted ACL reconstruction is limited in clinical practice due to several factors, including radiation exposure, the need for specialized training, and extended surgical duration^[^12^]^.

Computer navigation system has been developed to enhance the reproducibility and accuracy of surgical procedures, as well as to reduce the risk of tunnel positioning errors^[^13-15^]^. Raposo*, et al*^[^13^]^ introduced an MRI-based navigation system that offers detailed bone and soft tissue information. However, it has not been widespread due to its reliance on preoperative data collection. Guo*, et al*^[^16^]^ proposed an intensity-based 2D–3D registration navigation system for ACL reconstruction, which offers surgeons bone tunnel planning information based on both 2D and 3D data. While many studies have demonstrated that image registration and computer-assisted methods can improve the positioning accuracy of tunnels, most studies still manually locate the drill, which may compromise the accuracy of computer navigation system^[^13-16^]^. Therefore, the integration of navigation systems and surgical robotics can more accurately locate and navigate bone tunnels. Ding*, et al*^[^14^]^ reported the feasibility and accuracy of a surgical navigation robot for intraoperative navigation to locate the bone tunnel during ACL reconstruction using a bionic knee prosthesis. A preliminary cadaveric study confirmed the precision of bone tunnel drilling for ACL reconstruction surgery using a surgical navigation robot, demonstrating an accuracy of 1.8 mm ± 0.4 mm^[^17^]^. To date, there have been no reports on the clinical use of surgical navigation robot in ACL reconstruction surgery.

The purpose of the present study was to use a surgical navigation robot for anatomic ACL reconstruction to locate and navigate bone tunnels, and to compare it with a traditional handheld locator procedure. The efficacy and safety of the two groups were compared and analyzed, including the success rate of femoral tunnel positioning, tibial tunnel position, tunnel length, knee joint stability, and safety assessment. We hypothesized that the application of surgical navigation robot would result in a much higher accuracy in drilling bone tunnels with adequate safety.

## Materials and methods

### Trial oversight and design

This prospective, multicenter randomized study was conducted in four hospitals and involved 79 consecutive patients undergoing ACL reconstruction between April 2023 and November 2023. Institutional review board approval was obtained from each of the participating hospitals before the study commenced. All patients provided written informed consent before randomization. The work has been reported in line with the STROCSS criteria^[^18^]^.

### Patient population, randomization, and blinding

The inclusion criteria for this study included patients between the ages of 18 and 60 years who were skeletally mature and required unilateral ACL reconstruction with or without associated partial meniscectomy or meniscal repair. Patients with a history of previous knee surgery on the same side, multiligamentous injuries, revision surgery, or degenerative joint disease were excluded from this study. Basic patient demographic information and preoperative clinical data were collected and recorded, including age, sex, BMI, smoking, and time from injury to surgery. Preoperative evaluation of knee stability was conducted, involving the anterior drawer test, pivot shift test, and Lachman test.

After obtaining written informed consent, the research coordinator randomized patients to either surgical navigation robot group (robot group) or traditional handheld locator group (control group). A random number generator was employed to assign treatment to one of two groups: robot group or control group. Randomization was carried out at least 3 days before surgery to allow sufficient time for the surgical team to prepare the necessary equipment for the specified procedure in the operating room. All procedures in this study were performed by the same group of surgeons, thereby controlling for variability in skill level and experience. The surgeons involved in this study had extensive experience in traditional ACL reconstruction, with a minimum of 15 years of surgical practice. This experience was critical as prior studies suggested that higher levels of proficiency are correlated with improved surgical outcomes^[^19,20^]^. The surgeons underwent specific training programs for robot-assisted techniques, which were designed to shorten the learning curve associated with this novel approach^[^21^]^. This ensured that the potential discrepancies in performance due to varying levels of experience were minimized. Patients were unblinded to the surgical procedure they were scheduled to undergo. Investigators who conducted the statistical analysis were blinded. Following the completion of the analysis, the data were unblinded for the final interpretation of the results.

### Surgical procedures

With the patient in a supine position under general anesthesia, the surgeon conducted the pivot-shift test and the Lachman test to evaluate the stability of the knee joint. Standard anterolateral portal was established for arthroscopic inspection to determine the condition of the ACL and associated injuries. The meniscal lesions included 12 medial meniscus tears (eight in robot group and four control group), six lateral meniscus tears (two and four, respectively), and 18 involving both menisci (nine in each group). Partial/subtotal meniscectomy or meniscal repair were performed for meniscal tears. The gracilis and semitendinosus tendons were harvested, and each tendon is doubled over and sutured together to create a four-strand graft.

### Surgical navigation robot procedure

**Figure 1.**
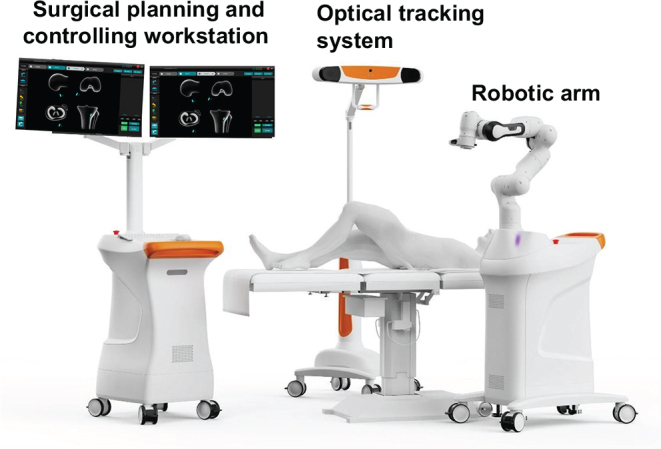
Composition of intelligent knee stability restoration.

An image-free technique using Intelligent Knee Stability Restoration (IKSR, Droidmed Medical Co., Ltd, Shanghai, China) robotic system in conjunction with arthroscopy was employed for the planning of bone tunnel position, intraoperative navigation, and tunnel drilling. The IKSR robotic system mainly consists of a surgical planning and controlling workstation, a robotic arm, and an optical tracking system (Fig. 1). The workstation facilitates the planning and adjustment of bone tunnel positions based on 3D model. The robotic arm has 7 degrees of freedom and can perform automatic navigation to precisely reach the location of the planned surgical access. The optical tracking device tracks the real-time spatial position of the patient’s knee.

**Figure 2.**
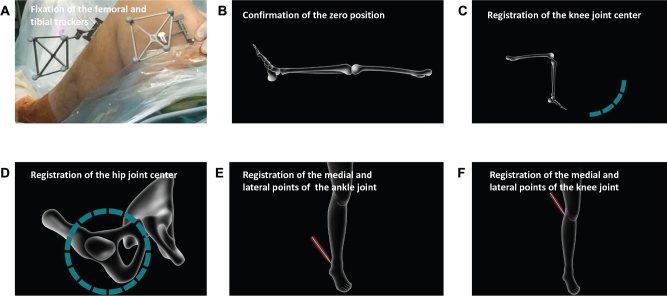
The preoperative planning process for creating knee models. (A) The femoral and tibial trackers were fixed to the medial tibia and lateral femur. (B) The knee joint was fully extended to confirm the zero position. (C) The femur was stabilized while the knee was flexed and extended to register its center. (D) The hip joint was stabilized, and the femur was rotated to register the center of the hip joint. (E) A locator was used to mark the medial and lateral points of the ankle joint. (F) A locator was used to mark the medial and lateral points of the knee joint.

**Figure 3.**
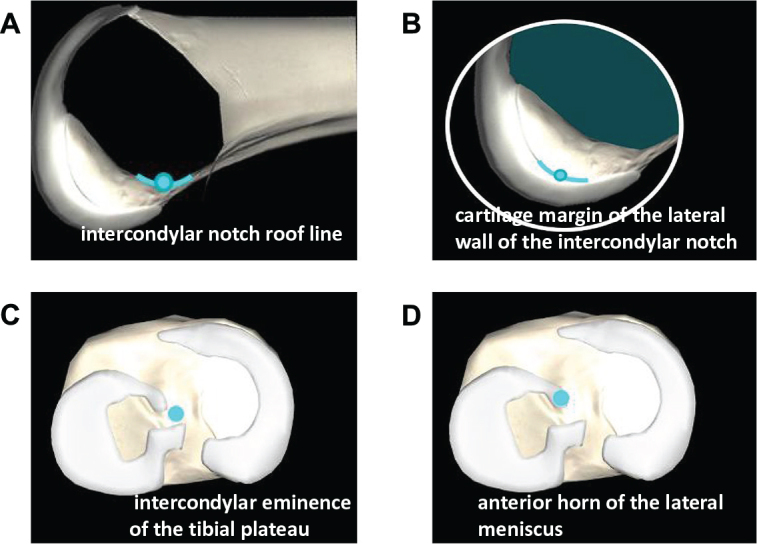
The planning of bone tunnel positions by identifying intra-articular reference points using a locator.

*Planning of bone tunnel position*: Firstly, the femoral and tibial trackers were fixed to the medial tibia and lateral femur, positioned approximately 15 cm away from the knee joint line, which can be tracked by the infrared tracker (Fig. 2A). The knee was fully extended to confirm the zero position and establish the reference point (Fig. 2B). Subsequently, the joint centers of the hip and knee were identified and established as per the instructions displayed on the workstation (Fig. 2C & 2D). Using a locator, the surgeon accurately marked the medial and lateral points of both the ankle and knee joints, thereby delineating the relative spatial relationships of the lower limb (Fig. 2E & 2F). For the single-bundle ACL reconstruction, inter-articular reference points were registered using a locator, including the intercondylar notch roof line, the cartilage margin of the lateral wall of the intercondylar notch, the intercondylar eminence of the tibial plateau, and the anterior horn of the lateral meniscus (Fig. 3). These inter-articular reference points were used to determine the anatomic position of the femoral and tibial tunnels, as Zhao proposed^[^22,23^]^. These steps are critical in facilitating the precise planning of the tunnel trajectory.

**Figure 4.**
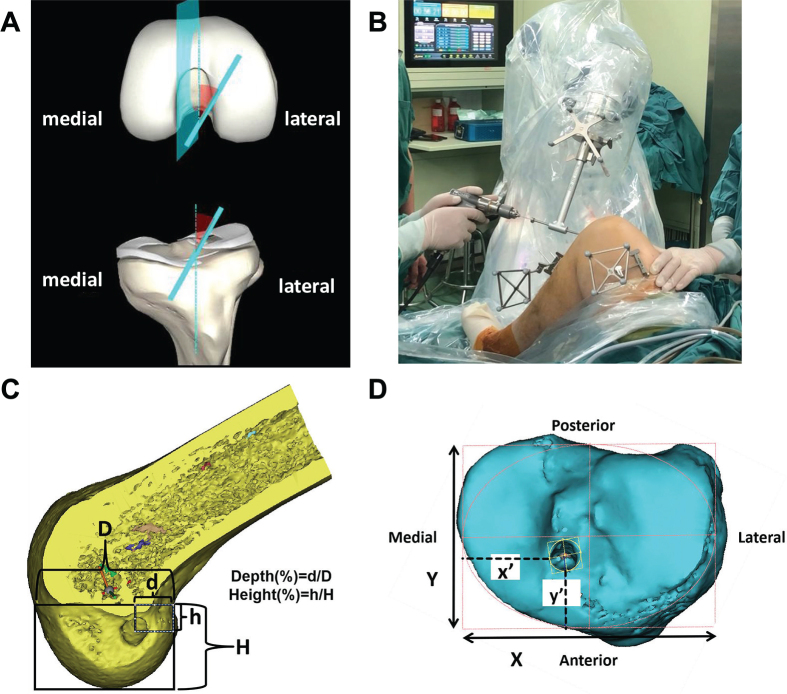
The intraoperative anterior cruciate ligament tunnel placement and assessment of intra-articular aperture positions using the quadrant method. (A) The planned pathways presented on the workstation. (B) Guided drilling of bone tunnels using automatic navigation of robotic arm. (C) The location of the center of the femoral tunnel aperture measured as a percentage of the depth and height of the lateral condyle. (D) The location of the center of the tibial tunnel aperture measured as a percentage of the total mediolateral and anteroposterior dimensions of the tibial plateau.

*Navigated drilling of bone tunnel*: Bone tunnels were drilled following the planned pathways using automatic navigation of robotic arm. In this process, it was crucial to ensure that both the trackers attached to the knee and the end of the robotic arm remained within the optical tracking system’s field of view. This ensured that the workstation can continuously monitor and obtain the spatial positions of both the knee and the robotic arm in real-time. The tibial and femoral tunnels were determined on the workstation, after which the foot pedal of the robotic arm was engaged to activate it. The robotic arm navigated accurately according to the workstation’s planned route, moving precisely to the target position (Fig. 4A). As the robotic arm moved, its position and the error between its current location and the planned tunnel route was displayed in real-time on the workstation. A cannula was then inserted, and the tunnel center was drilled using a 2.4 mm K-wire. The guide pin was passed from the tibia into the femur, forming a continuous line that connected the anatomic position of the tibial tunnel to the anatomic position of the femoral tunnel (Fig. 4B).

### Traditional handheld locator procedure

During all procedures, the ACL remnants were cleared to improve visualization during arthroscopy. Intra-articular reference points, including the inner edge of the anterior horn of the lateral meniscus and the intercondylar eminence of the tibial plateau, were used to identify the anatomic tibial insertion. The bony landmarks of the lateral intercondylar and bifurcate ridge were identified and utilized as reference points for femoral tunnel placement. After establishing the tibial tunnel, the guide pin was directed to the femur, and the femoral tunnel was drilled with the knee at 90° flexion. A rigid reamer matching the graft diameter was subsequently advanced over the guide pin and used to ream the bone tunnel. ACL graft passage and fixation were performed in standard fashion, using interference screw fixation on the tibia and cortical button fixation on the femur.

### Outcomes

The primary outcome was success rate of femoral tunnel positioning. One week after surgery, patients underwent three-dimensional computed tomography (3D CT). The position of the femoral tunnel was assessed using the quadrant method described by Bernard*, et al*^[^24^]^ (Fig. 4C). The position of the femoral tunnel center was measured as a percentage of the height and depth of the lateral condyle. A systematic review concluded the anatomic range of the ACL femoral footprint center, with the weighted 5th and 95th percentiles for anterior-posterior direction being 24% and 37%, and for proximal-distal direction being 28% and 43%^[^25^]^. The ideal position of the femoral tunnel was identified within this established normal range of the femoral footprint centers. The success rate specifically refers to the proportion of cases in which the femoral tunnel is placed accurately within the ideal anatomical position.

Secondary outcomes included tibia tunnel position, femoral and tibial tunnel lengths, knee stability, surgical time and safety. Femoral and tibial tunnel lengths were measured based on postoperative 3D CT, as was tibial tunnel position using the quadrant method as described by Bernard*, et al*^[^24^]^. (Fig. 4D). The position of the tibial tunnel center was measured as a percentage of the total anteroposterior and mediolateral dimensions of the tibial plateau^[^26^]^. One week after surgery, anterior drawer, Lachman’s, and pivot shift tests were conducted to evaluate the anterior and rotational stability of the reconstructed knee. Surgical time for each patient were meticulously recorded. The safety evaluation includes intraoperative and postoperative adverse events, defined as any unintended and undesirable outcomes that occur during or after the surgical procedure.

### Sample size calculation and statistical analysis

Based on our preliminary analysis, previous clinical findings, and expert opinions, the success rate of femoral tunnel positioning in control group was anticipated to be approximately 50%, while the robot group was expected to achieve a minimum success rate of 85%. With a power of 90% and alpha level of 0.025, the sample distribution ratio between the robot and control groups was set at 1:1. According to PASS 16.0 software, 33 cases were required in each group.

Comparisons between two groups were performed with the Student *t*-test for continuous normal distribution data (tibial tunnel position, femoral and tibial tunnel lengths, surgical time). The Pearson chi-square test was utilized for nominal categorical data (success rate of femoral tunnel positioning, knee stability). All statistical analyses were conducted using commercially available software (SPSS, SPSS Inc), with statistical significance set at *P* < 0.05.

## Results

**Figure 5.**
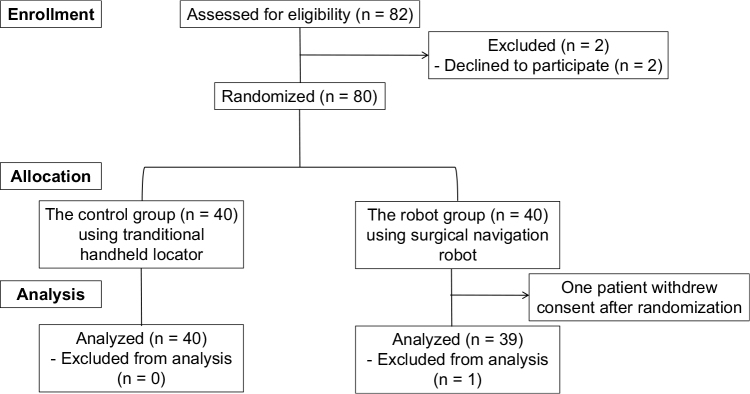
Consolidated Standards of Reporting Trials (CONSORT) flow diagram of the study.

From April 2023 through November 2023, a total of 80 patients were recruited and randomly assigned to the robot group (*n* = 40) or control group (*n* = 40) (Fig. 5). The distribution of patients across hospitals was as follows: hospital A recruited 12 patients for the control group (30%) and 12 for the robot group (30%), hospital B recruited six patients for each group (15%), hospital C recruited four patients for each group (10%), and hospital D recruited 18 patients for each group (45%). One patient in the robot group at hospital D withdrew consent after randomization without providing a reason. All of the remaining 79 patients included in the final analysis completed data collection. There were no statistically significant differences in any preoperative variables between the two groups including age, sex, BMI, or time from injury to surgery (*P* > 0.05) (Table 1). There were no significant differences in the preoperative evaluation of the anterior drawer test, Lachman test, and pivot shift test between the two groups (*P* > 0.05).

**Table 1:** Patient demographic

	Robot group (*n* = 39)	Control group (*n* = 40)
Age, M ± SD, y	36.1 ± 10.2	33.4 ± 13.0
Male sex, *n* (%)	27(69.2)	24(60.0)
BMI, M ± SD, kg/m^2^	24.7 ± 4.1	25.6 ± 4.2
Smokers%	7.7	0.0
Time from injury to surgery, M ± SD, wk	6.8 ± 4.3	7.4 ± 6.3

The success rate of femoral tunnel positioning in the robot group was significantly higher than that in the control group (82.1% vs 50.0%, *P* = 0.003) (Table 2). There was no significant difference between the two groups in the mean position of the tibial tunnel center in the anteroposterior direction (*P* = 0.301) and in the mediolateral direction (*P* = 0.217) (Table 2). There was no significant difference between the two groups in the lengths of the tibial tunnel (*P* = 0.163) and femoral tunnel (*P* = 0.478) (Table 2).

**Table 2:** Characteristics of tibial and femoral tunnel apertures, and surgical time

	Robot group (*n* = 39)	Control group (*n* = 40)	*P* value
Femoral aperture			
Anterior-posterior, M ± SD%	34.8 ± 6.9	32.1 ± 5.2	0.303
Proximal-distal, M ± SD%	34.3 ± 5.0	33.2 ± 7.3	0.614
Success rate %(*n*)	82.1(32)	50.0(20)	0.003*
Tibial aperture			
Anteroposterior, M ± SD%	43.3 ± 7.8	41.3 ± 8.8	0.301
Mediolateral, M ± SD%	45.4 ± 2.9	46.7 ± 1.4	0.217
Femoral tunnel length, M ± SD, mm	34.9 ± 5.2	35.7 ± 5.5	0.478
Tibial tunnel length, M ± SD, mm	35.3 ± 6.0	37.3 ± 6.6	0.163
Surgical time, M ± SD, min	122.8 ± 34.9	84.0 ± 28.3	<0.001*

^*^ indicates statistical significance (*P* < 0.05).

In terms of the anterior stability of the reconstructed knee, all patients exhibited negative results for both the anterior drawer test and Lachman test on the affected side postoperatively. Additionally, postoperatively, all patients demonstrated negative results for the pivot shift test. The surgical time in the robot group was significantly longer than that in the control group (122.8 min ± 34.9 min vs 84.0 min ± 28.3 min, *P* < 0.001) (Table 2).

A total of 17 patients experienced a total of 30 adverse events, as detailed in Table 3. Among them, eight patients in the robot group encountered 19 adverse events, while nine patients in the control group experienced 11 adverse events. The incidence rate of adverse events did not show statistical significance between the two groups (*P* = 0.830). There were no adverse events associated with the instruments or any serious adverse events. Additionally, no patients withdrew from the trial due to adverse events.

**Table 3:** Summary of adverse events observed during the study

	Robot group (*n* = 39)		Control group (*n* = 40)		*P* value
	No. of adverse events, *n*	No. of patients, *n*	No. of adverse events, *n*	No. of patients, *n*	
Dizziness	0	0	1	1	1.000
Incisional erythema	1	1	0	0	0.494
Muscle discomfort	5	3	1	1	0.590
Peripheral swelling	1	1	0	0	0.494
Constipation	4	4	1	1	0.340
Nausea	1	1	1	1	1.000
Abdominal discomfort	5	5	7	7	0.562
Palpitations	1	1	0	0	0.494
Thrombophlebitis	1	1	0	0	0.494
Total	19	8	11	9	0.830

## Discussion

In this multicenter randomized study, IKSR was utilized for locating and drilling bone tunnels during anatomical ACL reconstruction in clinical practice for the first time. Compared to a traditional handheld locator procedure, robot-assisted ACL reconstruction resulted in a higher success rate of femoral tunnel positioning. The results of tibial tunnel position, tunnel lengths, and postoperative knee stability in the robot group were similar to those in the control group. There were no adverse events associated with the instrument-related adverse events. However, the surgical time for robotic-assisted reconstruction was longer than that for the traditional handheld locator method.

The current findings demonstrated that the surgical navigation robot can enhance the accuracy of anatomical ACL reconstruction. In this study, the success rate was defined as the proportion of cases in which the femoral tunnel is placed accurately within the ideal anatomical position. This is achieved by combing with arthroscopy to visualize bony markers in the target area, employing infrared tracking reference markers for real-time positioning of the surgical area, and utilizing the robotic arm to guide the surgical path^[^13-15^]^. The optical tracking system navigates the surgical area using an infrared tracker in real-time to monitor the location of each section within the surgical area^[^14^]^. The robotic arm aids in accurate bone tunnel drilling, while the connected sleeve allows for precise positioning based on planned navigation^[^14,17^]^. This setup minimizes hand-induced shaking to ensure surgical accuracy and safety^[^14,17^]^. In terms of IKSR accuracy, a preliminary cadaveric study measured the distance between the planned and actual femoral tunnel position, revealing a mean difference was 1.8 mm ± 0.4 mm^[^17^]^. It indicates that the IKSR surgical navigation robot can achieve a predictable femoral tunnel position with high accuracy.

It has become increasingly evident that many aspects of the femoral tunnel are critical for achieving clinical success^[^27^]^. Therefore, there has been a growing focus on attaining an ideal femoral tunnel, especially in terms of aperture position. The present study demonstrated that robot-assisted ACL reconstruction resulted in a higher success rate of femoral tunnel positioning (82.1%) compared to a traditional handheld locator procedure (50.0%). The present study used the anatomic range of the ACL femoral footprint center (24%–37% for anterior-posterior direction and 28%–43% for proximal-distal direction) as a reference for identifying the ideal anatomical position^[^25^]^. Similarly, Dong*, et al*^[^28^]^ retrospectively analyzed data from 84 patients who underwent anatomic ACL reconstruction using the traditional handheld locator method, and found that 22 patients (26.2%) had their femoral tunnel positions within the ideal anatomical position. Compared to this study, the lower success rate of tunnel positioning in Dong’s study^[^28^]^ is attributed to the selection of only the midpoint of the bony ridge as the localization point, which may affect the accuracy of arthroscopic tunnel positioning. The control group in this study encountered several challenges that likely contributed to a success rate of only 50%. First, positioning the bone tunnel under arthroscopic guidance can be problematic due to visual bias, which may cause surgeons to misinterpret anatomical landmarks and, consequently, lead to inaccuracies in tunnel placement^[^7^]^. Additionally, the absence of navigation tools during the procedure can result in localization errors^[^13^]^. Furthermore, the use of hand-held locators inherently increases the risk of human error, making it difficult to drill the bone tunnels precisely along the planned angles and pathways. These limitations significantly contribute to the lower success rate observed in the control group. Furthermore, during the robot-assisted ACL reconstruction process, the angle of the tunnel can be visualized, enhancing the overall clarity of the procedure. Therefore, surgical navigation robots are invaluable for ensuring precise positioning of bone tunnels during anatomic ACL reconstruction surgery.

Epidemiologic data from the Multicenter ACL Revision Study showed that 37% of failed ACL reconstructions were attributed to incorrect tibial tunnel placement^[^29^]^. Cadaveric and clinical studies have demonstrated that the placement of the tibial tunnel can have a significant impact on the anterior knee stability after ACL reconstruction^[^30,31^]^. In the present study, the mean position of the tibial tunnel in the anteroposterior direction was 43.3% ± 7.8% in the robot group and 41.3% ± 8.8% in the control group. Parkar*, et al*^[^25^]^ concluded that the 5th and 95th percentiles of the tibial insertion center in the anteroposterior direction based on measurements in 300 knees were 39% and 46%. This indicates that both robot-assisted ACL reconstruction and traditional surgical methods can position the tibial tunnel within a normal range of native tibial ACL footprint.

The mismatch between graft length and tunnel length has been identified as a potential complication following ACL reconstruction, which could result in failure of interference screw fixation, graft extrusion, and reduced stiffness and laxity of the graft^[^32,33^]^. To prevent these issues, it is essential to ensure an optimal tunnel length. The mean femoral tunnel length in this study was found to be 35.3 mm ± 6.0 mm in the robot group and 37.3 mm ± 6.6 mm in the control group. A cadaveric study demonstrated that a femoral tunnel of less than 30 mm poses a higher risk of injuring the lateral collateral ligament, while a femoral tunnel longer than 30 mm has been found to be safe with respect to the articular cartilage^[^34^]^. Additionally, the mean tibial tunnel length in this study was found to be 34.9 mm ± 5.2 mm in the robot group and 35.7 mm ± 5.5 mm in the control group. Consistent with our results, Ko *et al*^[^32^]^ reported that the average length of the tibial tunnel was 33.7 mm using a traditional handheld locator method. Therefore, it can be concluded that both robot-assisted and traditional surgical methods for ACL reconstruction can create appropriate tunnel lengths for the femoral and tibial tunnels.

The residual anterior and rotational instability of the knee joint may lead to progressive osteoarthritic changes^[^35-37^]^. After ACL reconstruction, all patients in both the robot and control groups showed negative results for the anterior drawer test, Lachman test, and pivot shift test on the reconstructed-knee. Intraoperative and postoperative adverse events showed no significant differences between the robot-assisted and control groups. No intraoperative complications were directly associated with the surgical navigation robot system. However, the incidence of muscle discomfort was higher in the robot group, which may be attributed to the necessity of implanting rigid bodies during robotic surgery. Future research should focus on the development of non-implantable surgical navigation robots for ACL reconstruction, as this approach could help prevent adverse muscle healing and further enhance postoperative knee joint stability. No serious adverse events occurred, and no patients withdrew from the trial due to adverse events. However, the surgical time for robot-assisted ACL reconstruction (122.8 min ± 34.9 min) exceeded that of the traditional surgical method (84.0 min ± 28.3 min). This was primarily attributed to the surgeon’s extended planning time for placement and setup of surgical robot systems, as well as the additional time required for infrared tracer fixation. On the other hand, the surgeons lacked proficiency in robotic surgery techniques. It is anticipated that as surgeons become more adept in these surgical methods, the surgical time is likely to be significantly shortened. Despite the extended surgical time associated with robot-assisted ACL reconstruction, it has been noted that ACL reconstruction procedures typically take 2 hours or less^[^38^]^. This suggests that the additional time required for surgery when utilizing surgical navigation robots is within acceptable limits.

This study does have certain limitations. First, the correlation between knee stability over long-term follow-up periods remains unclear, warranting future clinical studies with extended observation periods. A second limitation is the lack of quantification of knee stability and kinematics for all included patients in this study. Manual stability testing is subjective and dependent on the surgeon’s expertise, potentially leading to results influenced by subjective judgment with limited accuracy and sensitivity. A third limitation is the lack of disaggregation of outcome data by gender. Future studies should consider a larger sample size to allow for a more in-depth analysis of outcomes based on gender. Lastly, this study only compared differences in femoral tunnel position and knee stability between robot-assisted and traditional surgical methods, without assessing postoperative knee function (e.g., knee function scale, long-term functional testing). Further research is warranted to provide additional clinical evidence supporting the future application of surgical navigation robots in ACL reconstruction surgery.

## Conclusions

Robot-assisted ACL reconstruction offers superior accuracy in drilling the femoral tunnel compared to the traditional handheld locator procedure. This method can drill the tibial tunnel and result in improved knee stability similar to the traditional surgical method. Although an increase in surgical time associated with surgical navigation robots has been noted, it is probable that the duration of surgery will be significantly reduced as surgeons gain proficiency in robotic procedures.

## Data Availability

The datasets used and/or analyzed during the current study are available from the corresponding author on reasonable request.

## Research registration unique identifying number (UIN)

The study was registered in the Chinese Clinical Trial Registry (ChiCTR-IOR-16009982).
